# Prevalence of lower urinary tract symptoms in a cohort of Australian servicewomen and female veterans

**DOI:** 10.1007/s00192-022-05254-x

**Published:** 2022-06-28

**Authors:** Simone D. O’Shea, Rod Pope, Katharine Freire, Robin Orr

**Affiliations:** 1 School of Allied Health, Exercise and Sport Sciences, Charles Sturt University, PO Box 789, Albury, NSW 2640 Australia; 2 Tactical Research Unit, Bond University, Robina, Australia; 3 Three Rivers Department of Rural Health, Charles Sturt University, Albury, Australia

**Keywords:** Military personnel, Urinary incontinence, Women, Occupational health, Genitourinary health, Pelvic floor dysfunction

## Abstract

**Introduction and hypothesis:**

Lower urinary tract symptoms (LUTS) are common in the general female population. It was hypothesised that Australian female military personnel and veterans would experience similar types and prevalence of LUTS as the broader Australian female population.

**Methods:**

An online cross-sectional survey was utilised to explore the pelvic health of active servicewomen and veterans in the Australian Defence Force (ADF). For the purposes of this report, only the demographic and LUTS data (excluding urinary tract infections) were extracted and descriptively analysed.

**Results:**

A total of 491 complete survey responses were received and analysed. Respondent characteristics were comparable to those documented in a departmental report regarding ADF servicewomen. No LUTS were reported by 38% of respondents. Regular symptoms of urinary incontinence were experienced by 27% of respondents (stress urinary incontinence = 23%, urge urinary incontinence = 16%, mixed urinary incontinence = 13%), bladder storage issues by 20–27%, and various voiding impairments by 9–27%. In addition, 41% reported regularly experiencing two or more LUTS, and for over two thirds of respondents, LUTS were an ongoing issue. Relationships between age, parity, and symptoms of urinary incontinence were also seen.

**Conclusions:**

Consistent with wider research in Australian female populations, LUTS were commonly experienced during service by ADF female military personnel and veterans. Given the high likelihood of female military personnel experiencing LUTS during their service, and a proportion experiencing ongoing symptoms, tailored monitoring and support for urinary health should be available to enhance occupational health, safety, and performance.

**Supplementary Information:**

The online version contains supplementary material available at 10.1007/s00192-022-05254-x.

## Background

Lower urinary tract symptoms (LUTS)—such as incontinence and bladder storage or voiding issues [1]—are more likely to occur in females than males [2–4]. It has been estimated that up to 80% of women experience LUTS. However, the frequency of symptoms and their impact on women’s wellbeing varies from mild to more severe [4]. Prevalence rates also vary across the lifespan between, and within, different types of LUTS [2, 3, 5]. For example, urinary incontinence, or involuntary leakage of urine [1], is one of the most commonly investigated sources of LUTS and reportedly affects between 13%–46% of Australian women [6, 7]. However, urinary incontinence is an umbrella term which encompasses a range of leakage sub-types, such as stress urinary incontinence (SUI, leakage during physical activity), urge urinary incontinence (UUI, leakage associated with urgency) and mixed urinary incontinence (MUI, leakage during physical activity as well as with urgency) [1]. The prevalence rates for these sub-types vary (for example, SUI 16%, UUI 7.5%, and MUI 18% in one sample of Australian women [8]), with SUI being more common in middle-age women and UUI in older women [8].

Negative physical, emotional, and social ramifications have been reported by women experiencing LUTS, such as reduced physical activity [9], anxiety, depression, and social isolation [10], reduced quality of life [11], and reduced work productivity [12]. Whilst further research is required to determine the relationships between different occupations and LUTS, a recent rapid review and meta-analysis found women engaged in more manual labour or physically demanding occupations had a higher risk of urinary incontinence [12]. In addition, women with LUTS have been found to alter behaviours associated with toileting and fluid intake at work [12] or modify work activities, such as physical training, to manage their symptoms [13]. These findings suggest a bi-directional relationship between LUTS and work.

Female military personnel are a growing occupational group. They typically engage in high levels of physical training and load carriage [14], often work in occupational contexts influencing fluid intake and voiding behaviours, and austere environments where sanitation may be problematic [15]. Equipment and workplace culture have also been reported to influence the risk of some types of LUTS and the self-care behaviours women use to prevent or manage urinary symptoms at work [16, 17]. Therefore, servicewomen may have an increased risk of LUTS and impaired occupational performance due to LUTS. A recent narrative review of pelvic floor health in female military populations found that approximately one third of US servicewomen experienced urinary incontinence [18]. However, no prevalence data were found for other LUTS (excluding urinary tract infections) or for women serving in the Australian Defence Force (ADF), which was the setting for the current study.

Consideration of the urinary health needs of female military personnel is important for enhancing their health, wellbeing, safety, and occupational performance. To inform potential mitigation and support strategies, it is necessary to understand the prevalence and characteristics of LUTS in Australian female military personnel. Therefore, the aims of this investigation were to determine the types, prevalence, and severity of LUTS experienced by ADF servicewomen and female veterans.

## Method

LUTS were explored in this study as part of a larger program of research on the pelvic health of Australian female military personnel and veterans. The research employed a cross-sectional design, using an online, anonymous survey. The survey approach had the advantage of being simple, efficient, and cost-effective to administer to a large sample from the chosen population [19, 20]. The survey was easily accessible to respondents regardless of their location, provided a robust description of the underlying population in relation to the topic area, and allowed for anonymous data collection to protect participant privacy and mitigate the risk of coercion to participate, or not, which is a particular ethical concern in military contexts [21]. Ethics approvals for the study were received from the Human Research Ethics Committees of the Departments of Defence and Veterans’ Affairs (099-19), Charles Sturt University (H19271), and Bond University (TCO1733). Data were collected between October 2019 and June 2020.

To be eligible to participate in the survey, individuals needed to identify as biologically female, be aged 18 years or over, and have actively served in the Australian Navy, Army, or Air Force for at least 6 months, in either a part- or full-time capacity. Due to the personal nature of pelvic health, potential participants were recruited through a combination of print (Navy, Army, and Air Force newspapers) and social media (Facebook) advertisements. This non-probability sampling recruitment strategy helped to mitigate the risk of coercion to participate, or not, which might have occurred if survey invitations were promulgated through on-site military establishments. It also enabled women to access information about the survey at a time and place that was comfortable, private, and secure for them. Prospective participants were provided with an information and consent statement on the survey landing page and had to click the ‘proceed’ button at the end of the page to indicate their understanding and consent prior to commencing the survey.

**Fig. 1:**
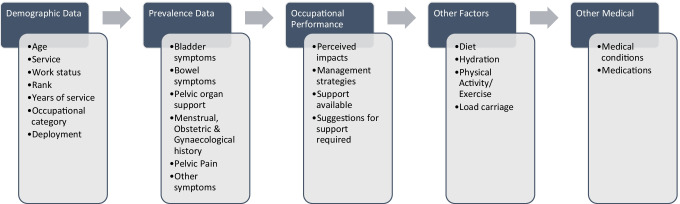
Sections and order of questioning within Pelvic Health Survey

Whilst many questionnaires exist for specific pelvic health issues, no previously validated survey instrument was identified to address the breadth of female pelvic health issues to be explored within the study. Therefore, a purpose-built questionnaire was developed by the research team for distribution via the Qualtrics survey platform. The survey questions were developed based on reports of prior research, consideration of the unique research aims and target population, and review of validated questionnaires that have been developed to assess different aspects of female genitourinary health and related issues [20, 22–27]. The online questionnaire utilised a standardised order of questions, which were presented in specific sections (Fig. 1). Skip logic was built into the survey design and allowed respondents to bypass questions that were not relevant to them, based on their responses to preceding questions [28]. The questionnaire included a range of open and closed question styles, including Likert and graphic rating scales, short-answer questions, and open narrative commentary boxes. The latter allowed participants the opportunity to elaborate on their personal experiences in the topic area [28]. The survey took participants on average 30–60 min to complete, depending on the number of questions that were relevant to them and the level of detail they provided. Within this study, only the demographic and prevalence data related to LUTS (excluding urinary tract infections) were analysed and are reported.

The survey development team consisted of four physiotherapists who had experience with women’s health and/or military populations, and a survey developer. Expert review of the survey instrument to critically assess and assure its functionality, content, language, context and relevance to the population, as well as its acceptability, was provided by: a senior Registered Nurse with extensive health care delivery experience within the military and public health setting; an Obstetrician and Gynaecologist with > 35 years of clinical experience; and an advisory group of active servicewomen (*n* = 10) from the Women’s Veterans’ Network Australia, representing each service (Navy, Army, Air Force). The survey was refined to address their feedback and recommendations.

Data on the following LUTS were collected within the elements of the survey considered in this report: urinary incontinence symptoms, including SUI, UUI, and MUI; bladder storage symptoms, including urinary urgency (UU) or sudden and pressing need to urinate; urinary frequency or the number of times respondents needed to urinate within an 8-h working shift; and nocturia or increased urine production overnight leading to interrupted sleep. Voiding symptoms, including incomplete bladder emptying, straining to empty the bladder, and weak urinary flow, as well as painful bladder emptying, were also explored. To avoid overinflating reported prevalence rates [5], only LUTS that occurred regularly (more than once per week) were counted when calculating prevalence estimates, and it was assumed that non-responses meant the participant did not experience the symptom.

All responses received were exported from the Qualtrics survey platform and imported into SPSS (Version 26) for analysis. Initial analysis was descriptive in nature, first estimating the response rate and demographic characteristics of respondents and then comparing them with previously reported population characteristics. Subsequently, measures of frequency and, where appropriate, indicators of the precision of population estimates were calculated for survey variables reflecting prevalence and severity of types of LUTS. Due to the size of the sample and concerns regarding family-wise error rates associated with numerous statistical tests, sub-group comparisons were limited and descriptive analysis used to identify trends within the data [28].

## Results

In total, 987 survey responses were received. However, the data from 496 survey responses were removed as they were incomplete (demographic data only). Therefore, a total of 491 survey responses remained to inform this study and were subsequently analysed.

The respondents were recruited from a population of ADF servicewomen and female veterans estimated to total 48,000, comprising approximately 13,600 active servicewomen [29] and an estimated 34,400 female veterans. In the absence of more precise data, the latter estimate was derived by doubling the number of female veterans who left military service in the 20-year period ending in 2019 (17,200 veterans [30]) to provide a crude estimate of the likely size of the entire Australian female veteran population in 2019. Based on this estimated total population size of servicewomen and female veterans in 2019, the survey sample of 491 respondents comprised approximately 1% of the underlying population. If the survey sample was representative of the underlying population and assuming a 95% confidence level, these figures would provide a margin of error for population estimates derived from the sample of ± 4%. However, the assumption of representativeness of the sample needed closer examination and is further considered below.

### Survey participation rate, sample characteristics, and representativeness of the sample

The survey participation rate was difficult to estimate. Assuming *all* ADF servicewomen and female veterans were reached by the survey invitation, as indicated above, the response rate would be approximately 1%. Clearly, not all eligible servicewomen and female veterans would have been reached, and so the actual participation rate will be higher but is unascertainable [31]. Nevertheless, if 10% of ADF servicewomen and female veterans were reached by the survey invitation, the participation rate would be approximately 10%, which remains relatively low and necessitates further assessment of the representativeness of the survey sample.

To assess the representativeness of the sample, the demographic profile of respondents was compared with the profiles of servicewomen obtained from the Defence Census 2019 [29] and Women in the ADF report 2017–2018 [32]. No similar data source for ex-serving Australian female military personnel exists. Therefore, the characteristics of this population are not known beyond their assumed similarity to currently serving women (though they will on average be older—the latter assumed because they were serving women before they were veterans). Thus, the demographic profile of veterans who responded to the survey was also compared to the profile of current servicewomen discussed above, while age differences were expected and assumed in the comparison.

The cohorts of respondents from the pelvic health survey were well matched to the servicewomen profiles reported [29, 32], across a range of attributes, once expected age differences for the veteran cohort were considered. Although the median age of our sample of active servicewomen was a few years higher than the median age of servicewomen reported in Defence Census data [29], when the proportions of servicewomen aged under and over 50 years in the survey sample and in the underlying population were compared, they were similar (Table 1). The distributions of women across Service arms were also comparable (Table 2), as were the median lengths of service for those currently employed full-time (Defence Census = 5 years, survey respondents = 6 years). However, a smaller proportion of active servicewomen who responded to the survey identified as Reservists (14%, 95% CI 10-19%) compared with Defence Census data indicating that 22% of female ADF personnel (2988 of 13,564) were employed in a part-time capacity [29].

**Table 1:** Age comparisons between survey respondents and Defence Census 2019 data

Comparison	Defence Census (2019)		Pelvic Health Survey			
			Actively serving (*n* = 299)		Veterans** (*n* = 192)	
	Full time	Reserve	Full time (*n* = 257)	Reserve (*n* = 42)	Full time (*n* = 178)	Reserve (*n* = 14)
Female Age (median years)	28	41	36	44	47.5	54
Age category						
< 50 years	91%	64%	193 (89%)	26 (67%)	86 (55%)	4 (36%)
50+ years	9%	35%	24 (11%)	13 (33%)	70 (45%)	7 (64%)

**Note: Median ages for female veterans (ex-serving women) were expected to be 10–20 years older than median ages for currently serving women and these findings are consistent with that expectation. The age profile for female veterans has not been compared with age data from the Defence Census (2019) because the survey only identified age of veterans at time of completing the survey, not during Service

**Table 2:** Comparison between survey respondents and Defence Census 2019 data for distributions of women across services

Service arm	Defence Census (2019)			Pelvic Health Survey				
	All women (*N* = 13,574)	Full time (n = 10,586)	Reserve (*n* = 2988)	All respondents (*N* = 491)	Actively serving (*n* = 299)		Veterans (*n* = 192)	
					Full time (*n* = 257)	Reserve (n = 42)	Full Time (n = 178)	Reserve (n = 14)
Navy	3428 (25%)	3026 (29%)	402 (14%)	105 (21%)	58 (23%)	4 (10%)	38 (23%)	5 (36%)
Army	6161 (45%)	4255 (40%)	1906 (64%)	258 (53%)	113 (44%)	31 (76%)	105 (59%)	9 (64%)
Air Force	3985 (29%)	3305 (31%)	680 (23%)	126 (26%)	85 (33%)	6 (14%)	35 (20%)	-

**Fig. 2:**
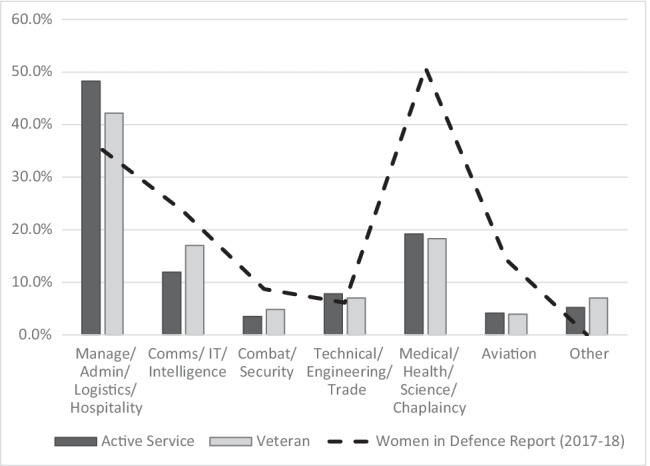
Comparison of ADF occupational categorisations between women participating in pelvic health survey and Women in Defence report 2017–2018.  Admin - administration; Comms - communications; IT - information technology. **Note: slight differences are noted between categories used in Women in Defence (2017-18) report and the survey. Within the pelvic health survey, women were also able to report more than one occupational category

A range of occupational groups exists within the ADF. Whilst there was slight variation in the terminology utilised, when comparing the pelvic health survey and Women in Defence report [32], Fig. 2 demonstrates similar patterns in the distributions of roles of women within the ADF.

To explore the sample for potential nonresponse bias—or, in other words, to appraise whether women without LUTS were potentially less likely to participate in the survey than women with symptoms—we compared the prevalence data for urinary incontinence in this survey with that reported in the literature for Australian women (12.8–46%) [6] as well as for female athletes (36%) [33] and military women (8–30%) [18]. Within the current pelvic health survey, SUI (> 1/week) was found to affect 23% of respondents (95% CI 19–27%) and UUI (> 1/week) 16% of respondents (95% CI 13–20%), with 27% of survey respondents (95% CI 22–30%) reporting at least one type of urinary incontinence and 13% (95% CI 10–17%) more than one (MUI). Despite the prevalence rates in the current survey falling within or below those reported in the broader literature, the potential for non-response bias remains.

Despite being a long questionnaire, strong completion rates for individual questions about prevalence were demonstrated throughout the survey (> 80%). Specifically, all questions associated with LUTS prevalence were answered by > 90% of survey participants (range: 90.8–93.5% question completion rate).

Additional characteristics of the survey cohort (Table 3) demonstrate that respondents had a wide range of service experience, were of varied ranks, and had actively participated in field and deployment opportunities. Details are also provided about their health and regular medication use.

**Table 3:** Demographic data for respondents, as entire cohort and by current activity status

Participant attributes	All respondents (*n* = 491)	Activity status	
		Actively serving (*n* = 299)	Veteran (*n* = 192)
Mean (range) Age (years)	42 (19–78)	38 (19–63)	48 (20–78)
Service arm			
Navy	105 (22%)	62 (21%)	43 (22%)
Army	258 (53%)	144 (48%)	114 (59%)
Air Force	126 (26%)	91 (30%)	35 (18%)
Service years			
< 10 years	191 (39%)	94 (31%)	97 (51%)
10–19 years	167 (34%)	112 (38%)	55 (29%)
> 20 years	133 (27%)	93 (31%)	40 (21%)
Rank			
Commissioned Officer	172 (35%)	138 (46%)	34 (18%)
NCO/WO*	167 (34%)	98 (33%)	69 (36%)
Other rank	142 (29%)	59 (20%)	83 (43%)
Cadet/Trainee/Recruit	10 (2%)	4 (1%)	6 (3%)
Participated in field activities	397 (81%)	241 (81%)	156 (81%)
Experienced deployment	342 (70%)	236 (79%)	106 (55%)
Australia	166 (34%)	112 (38%)	54 (28%)
Overseas	287 (59%)	208 (70%)	79 (41%)
Experienced pregnancy			
Yes	281 (57%)	164 (55%)	117 (61%)
No	114 (23%)	78 (26%)	36 (19%)
Unspecified	96 (20%)	57 (19%)	39 (20%)
Health during service			
Nil issues	165 (34%)	108 (36%)	57 (30%)
Back/hip pain	315 (64%)	192 (64%)	123 (64%)
Other musculoskeletal issues	76 (16%)	37 (12%)	39 (20%)
Respiratory conditions	96 (20%)	47 (16%)	49 (26%)
BMI > 25	122 (25%)	79 (26%)	43 (22%)
Neurological conditions	11 (2%)	5 (2%)	6 (3%)
Metabolic conditions	6 (1%)	3 (1%)	3 (2%)
Inflammatory conditions	27 (6%)	10 (3%)	17 (9%)
Vascular conditions	7 (1%)	2 (1%)	5 (3%)
Psychological conditions	88 (18%)	42 (14%)	46 (24%)
Eating disorder	6 (1%)	4 (1%)	2 (1%)
Food allergy/intolerance	82 (17%)	54 (18%)	28 (15%)
Other	7 (1%)	2 (1%)	5 (3%)
Medications (regular use in service)			
No medications	101 (21%)	67 (22%)	34 (18%)
Antihistamines	72 (15%)	42 (14%)	30 (16%)
Blood pressure	12 (2%)	7 (2%)	5 (3%)
Anti-inflammatory	141 (29%)	86 (29%)	55 (29%)
Anti-depressants	50 (10%)	24 (8%)	26 (14%)
Anti-psychotics	3 (1%)	1 (0.3%)	2 (1%)
Sleeping tablets	28 (6%)	17 (6%)	11 (6%)
Strong pain medications	80 (16%)	36 (12%)	44 (23%)
Antibiotics	59 (12%)	19 (6%)	40 (21%)
Smoker (during service)	61 (12%)	26 (9%)	35 (18%)

*NCO/WO: non-commissioned officer/warrant officer; BMI: body mass index

### Survey findings

Over one third of participants (*n* = 186, 38%) reported that they did not regularly experience any of the LUTS included within the survey. Table 4 provides a breakdown of the prevalence data for servicewomen and female veterans not experiencing specific LUTS.

**Table 4:** Prevalence of ADF servicewomen and female veterans not experiencing LUTS during active service

No reported symptoms	All respondents (*n* = 491)	Actively serving (*n* = 299)	Veteran (*n* = 192)
Urinary continence			
No reported SUI	136 (28%)	86 (29%)	50 (26%)
No reported UUI	178 (36%)	114 (38%)	64 (33%)
No reported MUI	210 (43%)	136 (46%)	74 (39%)
Bladder storage			
Urinary frequency (< 5/shift)*	339 (69%)	203 (68%)	136 (71%)
No urinary urgency	138 (28%)	90 (30.1%)	48 (25%)
No nocturia	391 (80%)	239 (80%)	152 (79%)
Voiding			
No emptying concerns	360 (73%)	223 (75%)	137 (71%)
No bladder straining	445 (91%)	273 (91%)	172 (90%)
No urinary flow concerns	425 (87%)	261 (87%)	164 (85%)
Pain			
No bladder pain emptying	430 (88%)	273 (91%)	157 (82%)

*SUI*, stress urinary incontinence; *UUI*, urge urinary incontinence; *MUI*, mixed urinary incontinence. *Normal urinary frequency defined for purposes of prevalence estimates as the need to urinate fewer than five times within an 8-h working shift

The prevalence data for LUTS are presented in Table 5 and demonstrate that a variety of LUTS were commonly experienced by women during their military service. Within this cohort of servicewomen and female veterans, 21% of respondents (*n* = 105) reported regularly experiencing one LUTS, and 41% (*n* = 200) regularly experienced two or more LUTS. A similar pattern of prevalence during service for each symptom, other than UUI, was observed for active servicewomen and veterans. Therefore, combined data are reported in further analyses.

**Table 5:** Prevalence of regular LUTS during service in the surveyed cohort of ADF servicewomen and female veterans

LUTS	All respondents (*n* = 491)	Actively serving (*n* = 299)	Veteran (*n* = 192)
Urinary incontinence			
SUI (> 1/week)	112 (23%)	61 (20%)	51 (27%)
UUI (> 1/week)	78 (16%)	32 (11%)	46 (24%)
MUI (> 1/week)	57 (13%)	22 (7%)	35 (20%)
Bladder storage			
Urinary frequency (5+/shift)*	120 (24%)	74 (25%)	46 (24%)
Urinary urgency (> 1/week)	131 (27%)	76 (25%)	55 (29%)
Nocturia	100 (20%)	60 (20%)	40 (21%)
Voiding			
Incomplete emptying	131 (27%)	76 (25%)	55 (29%)
Bladder straining	46 (9%)	26 (9%)	20 (10%)
Weak urinary flow	66 (13%)	38 (13%)	28 (15%)
Pain			
Painful bladder emptying	61 (12%)	26 (9%)	35 (18%)

*SUI*, stress urinary incontinence; *UUI*, urge urinary incontinence; *MUI*, mixed urinary incontinence. Urinary frequency defined for purposes of prevalence estimates as the need to urinate five or more times within an 8-h working shift

**Fig. 3:**
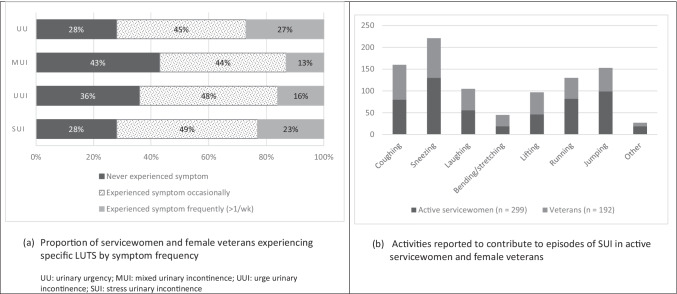
Prevalence of specific lower urinary tract symptom frequency (**a**) and activities contributing to symptoms of SUI (**b**)

Almost one quarter of respondents (23%, 95% CI 19–27%) reported they experienced SUI more than once per week during service (Fig. 3a). However, when including all women who experienced episodes of SUI even ‘occasionally’ or ‘sometimes’ during activities at work, SUI affected up to 63% of respondents (95% CI 58–67%). The volume of leakage reported during episodes of SUI was most commonly ‘slightly wet underwear, liner or pad’ (49%) or ‘a dribble’ (42%). However, for one in every ten women who reported SUI, episodes of leakage were reported to require a complete change of their underwear, pad, or clothing. The most common activities contributing to episodes of SUI were those involving an increase in intra-abdominal pressure (e.g., sneezing, coughing) and vertical ground reaction force loading (e.g., running, jumping) (Fig. 3b). Similarly, only 16% (95% CI 13–20%) and 13% (95% CI 10–16%) of respondents experienced regular episodes of UUI or MUI, respectively (Fig. 3a), but this increased to 55% (95% CI 50–59%) and 53% (95% CI 48–57%), when occasional episodes were included in the prevalence rate.

Many servicewomen and female veterans reported that their LUTS were “current and ongoing issues”. LUTS such as SUI, UUI, and UU were reported to be ongoing issues for two thirds of active servicewomen (SUI = 66% 95% CI 60–71%; UUI = 63% 95% CI 57–68%; UU = 69% 95% CI 63–74%), and over three quarters of veterans (SUI = 76% 95% CI 70–82%; UUI = 82% 95% CI 76–87%; UU = 78% 95% CI 72–83%). As expected, due to the older age of the veteran cohort, it was more common for veterans to report they had experienced LUTS for a longer duration, commonly in excess of 10 years (supplementary data).

**Fig. 4:**
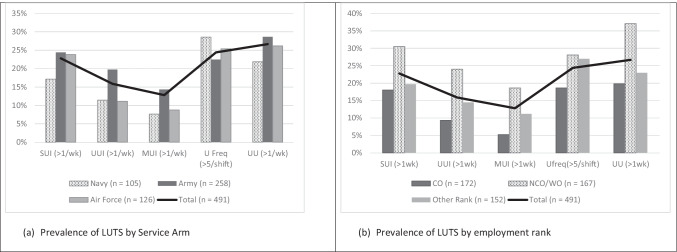
Urinary symptoms for all respondents presented by (**a**) service arm and (**b**) employment rank. SUI - stress urinary incontinence more than once per week; UUI – urge urinary incontinence more than once per week; MUI- mixed urinary incontinence more than once per week; UFreq - urinary frequency greater than five times during an eight-hour shift; UU - urinary urgency more than once per week; CO – commissioned officer; NCO/WO – non-commissioned officer/warrant officer

**Fig. 5:**
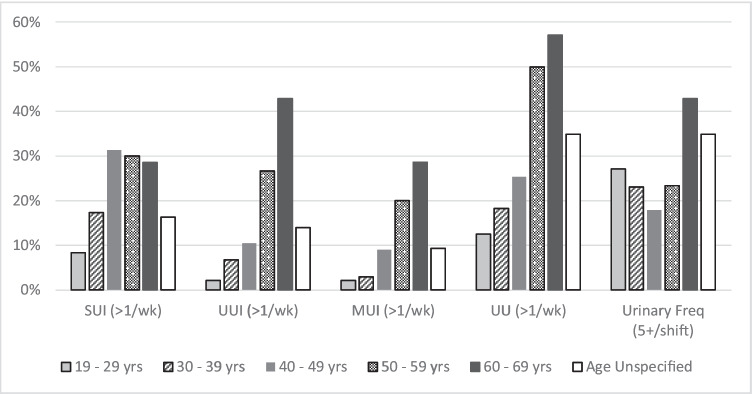
Prevalence of LUTS in active servicewomen by age category**. SUI - stress urinary incontinence more than once per week; UUI – urge urinary incontinence more than once per week; MUI- mixed urinary incontinence more than once per week; UFreq - urinary frequency greater than five times during an eight-hour shift; UU - urinary urgency more than once per week. **Age category reporting is not presented for female veterans, as their age during last period of active service was not collected – only their age at time of completing the survey

**Fig. 6:**
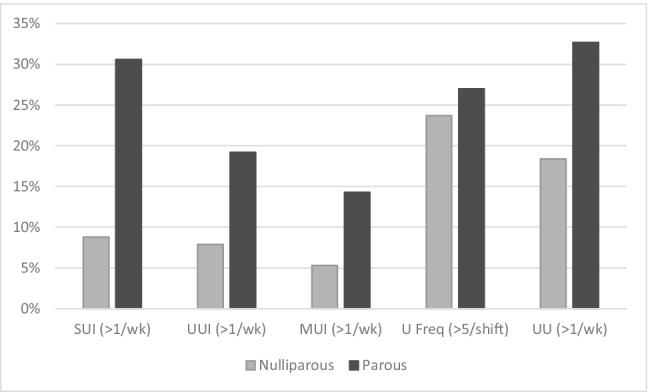
History of pregnancy and prevalence of urinary symptoms. SUI - stress urinary incontinence more than once per week; UUI – urge urinary incontinence more than once per week; MUI- mixed urinary incontinence more than once per week; UFreq - urinary frequency greater than five times during an eight-hour shift; UU - urinary urgency more than once per week; Nulliparous – respondents who reported never being pregnant; Parous – respondents who reported being pregnant one or more times

Comparisons of LUTS prevalence rates by service arm, employment rank, age group, and parity groups are presented in Figs. 4, 5, and 6. A similar pattern of prevalence was seen for all urinary symptoms, except UUI (and as a result, MUI), in service comparisons (Fig. 4a), whereas a trend towards higher prevalence rates of UI and UU symptoms were reported by women in Non-Commissioned Officer/Warrant Officer ranks (Fig. 4b). In active servicewomen, symptoms of UUI and UU increased in prevalence with each decade of increasing age, whereas SUI prevalence increased up until mid-life (40–49 years) and then stabilised (Fig. 5). Figure 6 demonstrates a clear relationship between having a history of pregnancy and the experience of symptoms such as SUI, UUI, MUI, and UU.

Additional comparisons were targeted to variables that may have previously demonstrated a relationship with LUTS and had reasonable sub-group sample sizes (BMI [34], respiratory conditions [35], and lower back/hip pain [36, 37]). The prevalence of bladder storage, voiding, and pain symptoms were higher in servicewomen and veterans also reporting regular episodes of lower back/hip pain, but no clear trends were seen for the prevalence of LUTS in women associated with higher BMI or respiratory conditions (supplementary data).

## Discussion

This was the first known study to explore the prevalence of LUTS in a cohort of Australian female military personnel and veterans. Approximately 1 in every 100 current female personnel and veterans responded, with the profile of respondents similar to the population characteristics of female ADF personnel reported in the Women in the ADF Report 2017–2018 and Defence Census 2019. The survey found regular LUTS were common for female personnel during active military service, and the patterns of LUTS were similar to those reported in the broader female population [6–8].

Australian servicewomen and female veterans commonly reported experiencing one or more LUTS during their military service. Urinary incontinence symptoms of different types affected 13–23% of these women, bladder storage symptoms 20–27%, voiding symptoms 9–27%, and bladder emptying pain 12% of respondents. Prevalence statistics for LUTS in the general female population are frequently reported for symptoms of urinary incontinence, and the rates identified within this survey among Australian military women and female veterans are consistent with those for Australian women more broadly [6–8]. However, it is important to recognise that prevalence rates are influenced by the definitions used and need to be interpreted in that context. To prevent overinflating prevalence statistics [5], within this study primary calculations of prevalence rates were based on numbers of women reporting consistent and frequent symptoms (more than once/week). However, when all levels of symptom experience were considered, LUTS were reported to occur in between 50–70% of respondents (depending on symptom type). Whilst these rates do not provide insight to the level of impact on well-being or occupation, they do highlight that it is highly likely that female personnel will experience one or more types of LUTS at some point during their military service and that at least one fifth will experience LUTS of some type consistently.

Similar to the general female population and previous studies of female military personnel [13], age and parity status were associated with prevalence rates of LUTS in the military women and female veterans who responded to this survey—particularly symptoms of urinary incontinence. Consistent with findings reported in the broader literature, SUI was more common in younger women, with prevalence peaking at mid-life, whereas UUI rates were shown to steadily increase with age [5, 8]. Recognising the influence that age and parity may have on LUTS highlights that female military personnel may have different genitourinary health risks and needs at varying times within their period of military service. Other research has similarly demonstrated there is variation in the rates of occurrence of different types of LUTS experienced by females across their lifespan [3]. These findings suggest that genitourinary health assessment, monitoring, and support provided to servicewomen should be tailored to specific needs of each servicewoman during their time in service and on separation. Examples of such health care could include screening programmes and education at time of recruitment, post-partum return to work planning, pre-deployment preparation, and support around menopause. Adequate and tailored support may contribute to reducing rates of separation that may be potentially occurring from LUTS that are negatively impacting on occupational performance.

Occupational elements altering bladder behaviours at work have been suggested as potential factors influencing the prevalence of female LUTS [12]. Despite being employed in varied occupational roles, all military personnel are required to undertake regular organised and mandated physical and military training. However, the fitness requirements for each service are different, the duration and frequency of physical loading will vary between roles, military personnel may engage in multiple roles throughout their service, and the workplace environment also changes depending on the posting. Therefore, the influence of military occupational factors on the prevalence of female LUTS is likely to be challenging to determine. Within this study, similar patterns of LUTS prevalence were observed for Naval, Army, and Air Force women, suggesting that the Service arm was not a factor influencing LUTS. Contrarily, employment rank may have a relationship with LUTS, as a trend towards higher urinary incontinence and urgency prevalence rates was reported for female Non-Commissioned Officers/Warrant Officers. It is likely that confounding variables, such as age, parity, and length of service, may contribute to this finding, as progression through the ranks is likely to take some time.

For the majority of servicewomen and female veterans reporting LUTS, their symptoms were identified to be “current and ongoing”. Whilst active servicewomen with LUTS were more likely to report that they had had their symptoms < 6 years, close to one in five active servicewomen and almost half the female veterans with LUTS reported symptom durations > 10 years. These findings concur with longitudinal studies of women with urinary incontinence, which have found that symptoms can persist and fluctuate significantly, with periods of remission, and reduced or increased symptoms [5]. The presence of persistent LUTS reinforces the potential benefits of targeted support for servicewomen to prevent the cumulative effects of long-term LUTS on occupational performance. However, it must be acknowledged that the survey questions did not explore the level of symptom fluctuation experienced by respondents or remission or cure rates within the cohort.

The frequencies of LUTS experienced by servicewomen and female veterans provide insight into the severity of the problem, identifying that a substantial number of servicewomen experience symptoms more than once per week. In addition, other dimensions of symptom characteristics provide insight into severity. For example, the amount of urinary leakage in those experiencing incontinence is another indicator. Consistent with previous research involving women with urinary incontinence [7], most affected respondents reported mild (a “dribble”) to moderate (“slightly wet underwear”) levels of leakage. Despite most experiencing mild to moderate symptoms, the occupational impacts of these symptoms are not fully reflected in the prevalence data. LUTS have been reported to reduce work productivity [12] and reduce female physical exercise participation rates, with those who experience severe symptoms being more likely to cease exercise [38]. Therefore, even mild to moderate symptoms may present challenges for females in military contexts, such as in the field or on deployment, and may influence safety and work performance. For example, servicewomen with urinary incontinence have been found to manage symptoms through fluid restriction and altering their voiding patterns [15], which may increase their risk of developing heat related illness. The prevalence findings from this study highlight that urinary health should be monitored, and personnel offered support to maintain and manage it at work.

The findings presented here provide useful understanding of the types, prevalence, and severity of LUTS in Australian servicewomen and female veterans, but the data presented do not provide insights into the impacts of LUTS for female personnel within occupational settings, how servicewomen manage LUTS in military work environments, the influence of symptoms on occupational performance, or the military support available and utilised by servicewomen. Due to the importance of these questions and gaining more in-depth understanding of the impact of pelvic health more broadly (not just limited to LUTS) on the occupational experiences of servicewomen, data from the pelvic health survey associated with work performance are not presented within this report.

Whilst the findings of the survey provide useful insights into LUTS for female military personnel within Australia, it is important to acknowledge limitations of the study. Self-report surveys can be impacted by recall bias, which was a particular concern for the cohort of female veterans due to their need to remember historical LUTS, experienced during their last period of active service. The original study criteria excluded female veterans from participating in the survey for this reason. However, feedback was received from the servicewomen consulting on the survey that the female veteran population strongly wanted to participate and share their experiences. Despite this limitation, the similarities in prevalence rates for LUTS reported by servicewomen and female veterans suggest that recall bias may not have had a substantial impact on the findings of the survey. Due to the breadth of the pelvic health survey, completion of the survey was time-consuming, and this may have contributed to the large number of incomplete survey responses needing to be excluded from data analysis. This increased the potential for non-response bias or systematic differences in LUTS between those who did and did not respond to the survey. Consultation with potential participants occurred during survey design to ensure the survey length was acceptable, and data have been presented to allow judgements on the representativeness of the sample to be considered. Finally, generalisation of the survey findings should continue to be done with caution because of the use of non-probability sampling methods [31].

## Conclusion

The survey found that LUTS were commonly experienced by ADF female military personnel during service and that the types of LUTS, their prevalence rates, and the levels of severity of symptoms were similar to those reported in Australian females more broadly. Consistent with wider research, relationships between LUTS and age and parity were also demonstrated in this cohort of servicewomen and female veterans. Given that LUTS may impact on occupational performance and that most ADF servicewomen are likely to experience LUTS at some time during their service, with a proportion experiencing ongoing symptoms, tailored monitoring and support services should be available to servicewomen. These support systems will need to vary in type across the period of service for each individual and in response to variations in work context.

## Supplementary information

ESM 1 (PDF 203 kb)
