# Supplementary material for: Prevalence of lower urinary tract symptoms in a cohort of Australian servicewomen and female veterans
Source: Int Urogynecol J. 2022 Jun 28;34(4):885–96. doi: 10.1007/s00192-022-05254-x (PMC10038961; doi:10.1007/s00192-022-05254-x)
Supplement: Supplementary file 1 — (PDF 203 kb) [file 192_2022_5254_MOESM1_ESM.pdf]

Figure S1: Duration of LUTS reported by active servicewomen and female veterans

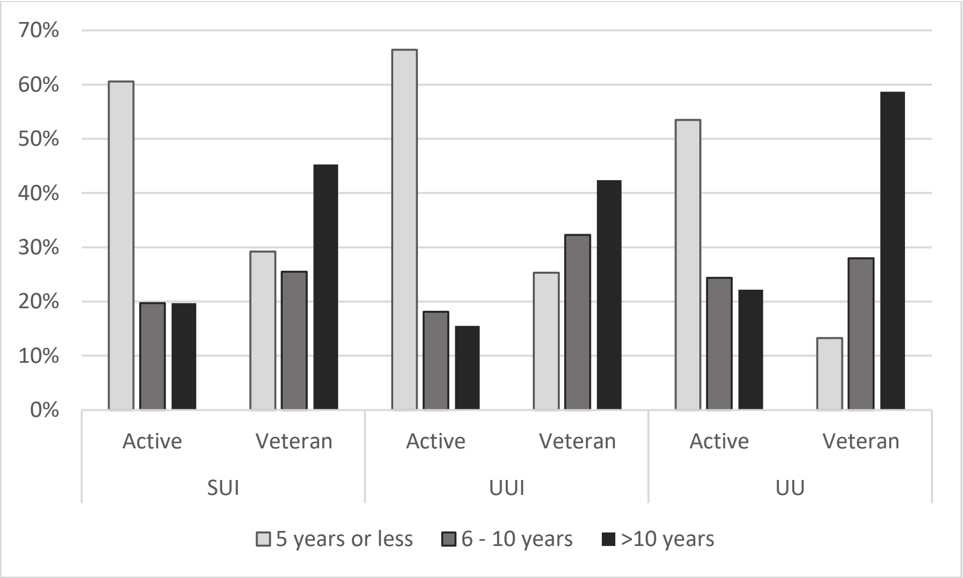

SUI – stress urinary incontinence; UUI – urge urinary incontinence; UU – urinary urgency

Figure S2: LUTS prevalence by body mass index (greater than or less than 25)

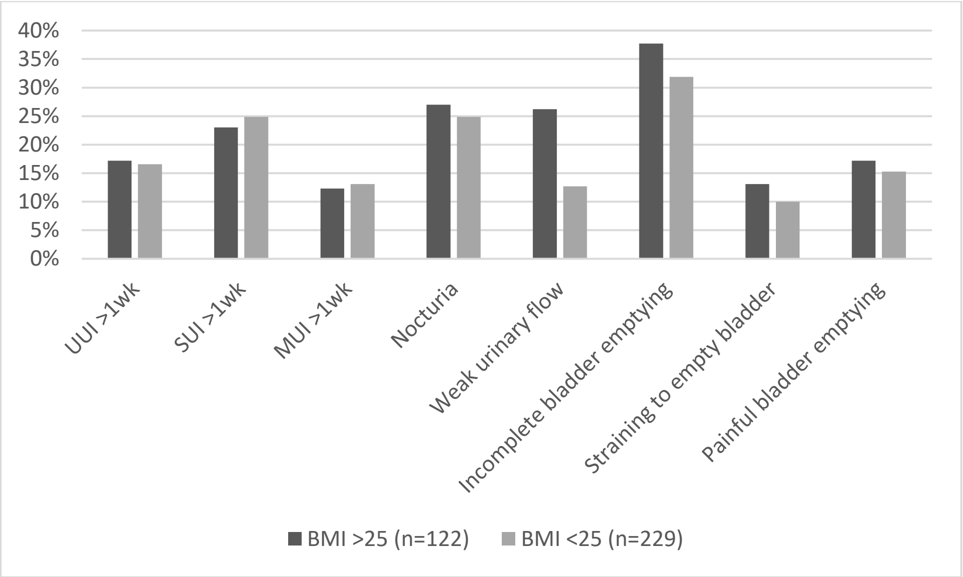

BMI: body mass index

Figure S3: LUTS prevalence by presence or absence of respiratory condition

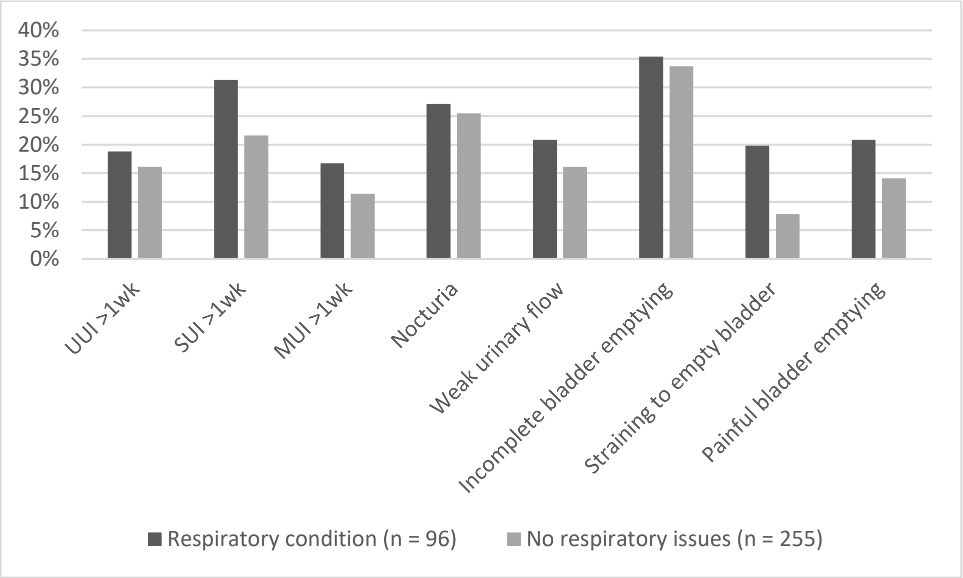

Figure S4: LUTS prevalence by presence or absence of lower back/hip pain

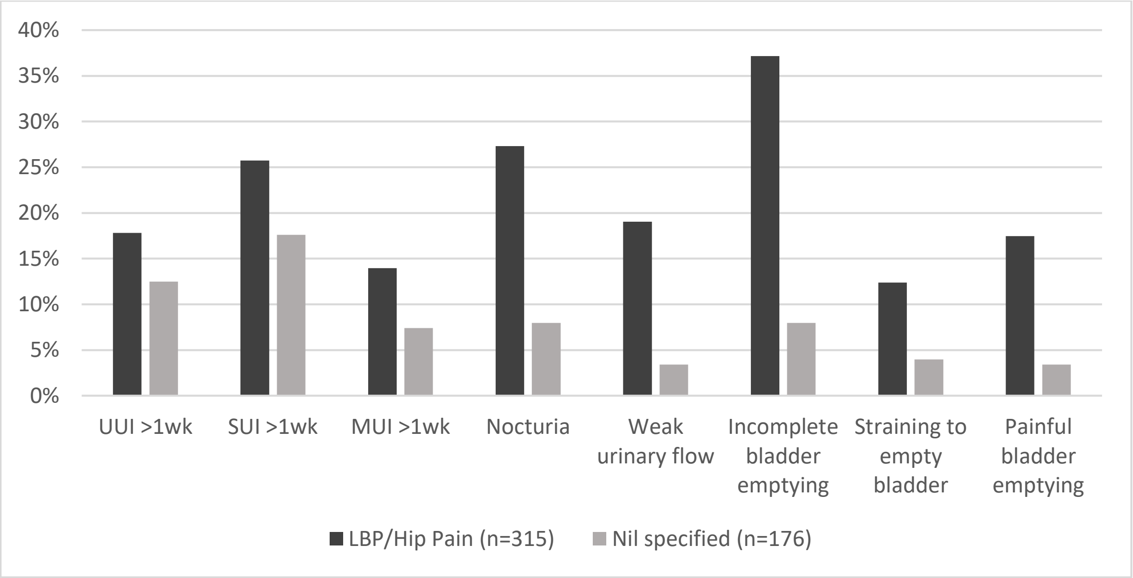

LBP: lower back pain
